# The UWHAM and SWHAM Software Package

**DOI:** 10.1038/s41598-019-39420-x

**Published:** 2019-02-26

**Authors:** Bin W. Zhang, Shima Arasteh, Ronald M. Levy

**Affiliations:** Center for Biophysics and Computational Biology, Department of Chemistry and Institute for Computational Molecular Science, Temple University, Philadelphia, Pennsylvania 19122 United States

## Abstract

We introduce the UWHAM (binless weighted histogram analysis method) and SWHAM (stochastic UWHAM) software package that can be used to estimate the density of states and free energy differences based on the data generated by multi-state simulations. The programs used to solve the UWHAM equations are written in the C++ language and operated via the command line interface. In this paper, first we review the theoretical bases of UWHAM, its stochastic solver RE-SWHAM (replica exchange-like SWHAM)and ST-SWHAM (serial tempering-like SWHAM). Then we provide a tutorial with examples that explains how to apply the UWHAM program package to analyze the data generated by different types of multi-state simulations: umbrella sampling, replica exchange, free energy perturbation simulations, etc. The tutorial examples also show that the UWHAM equations can be solved stochastically by applying the RE-SWHAM and ST-SWHAM programs when the data ensemble is large. If the simulations at some states are far from equilibrium, the Stratified RE-SWHAM program can be applied to obtain the equilibrium distribution of the state of interest. All the source codes and the tutorial examples are available from our group’s web page: https://ronlevygroup.cst.temple.edu/software/UWHAM_and_SWHAM_webpage/index.html.

## Introduction

The weighted histogram analysis method (WHAM) algorithm^1,2^ is widely applied to estimate the density of states and free energy differences based on the data generated by multi-state simulations. Multi-state simulations are popular advanced sampling algorithms that are applied in computational biophysics and computational chemistry. For example, the temperature replica exchange method is extensively applied to explore the configurational space of biomolecules; the umbrella sampling method is applied to construct free energy landscape of a system on chosen reaction coordinates; the free energy perturbation and Hamiltonian replica exchange method are powerful tools used to estimate the binding affinities of ligands and proteins for small-molecule drug discovery^3–5^. The WHAM algorithm is the standard tool to analyze the data generated by these multi-state simulations. Consider the simulation at each state as a measurement of density of states, the WHAM algorithm answers the question what the best estimate of density of states is if measurements have been taken at multiple states.

Since its introduction in 1992, the WHAM algorithm has been examined and studied by several research groups^6–11^. The most important improvement of WHAM is that an unbinned WHAM version named the multi-state Bennett acceptance ratio (MBAR) or the binless WHAM (UWHAM) was introduced^12–14^. Compared with the original WHAM, which coarse-grains observations into bins of a histogram, the binless WHAM provides the estimate of density of states for each data point therefore increasing the statistical precision and importantly, estimating the density of states provides a connection with the potential distribution theorem^15,16^.

Complementary to the study of WHAM itself, how to solve WHAM equations efficiently in practice is another topic that has been an object of research^17–20^. In fact, this topic became more challenging and more urgent after the introduction of binless WHAM because of the dramatic increase of the number of variables without coarse-graining. In ref.^14^, Tan *et al*. proposed to solve the UWHAM equations by minimizing a convex function. To further remove the computational bottleneck in scaling up UWHAM, we developed methods called stochastic UWHAM (SWHAM) which solve the UWHAM equations stochastically by using generalized ensemble algorithms to resample the data collected at multiple states^21,22^. One important assumption of applying WHAM is that the data obtained from each state has already reached global equilibrium. However, sometimes this assumption does not hold if the barriers between free energy basins are high at some of the states and the simulation times are not long enough. We developed a method called Stratified-UWHAM^23^ to analyze the data generated by multi-state simulations when the simulations at some states are far from equilibrium.

The purpose of this paper is to introduce the UWHAM and SWHAM software package developed by our group. The programs used to solve the UWHAM equations are written in the C++ language and operated via the command-line interface. The basic solver solves the UWHAM equations by either a direct iteration method or minimization of a convex function. When the data ensemble is large, we show that the multi-state free energies can be obtained directly by running serial tempering-like SWHAM (ST-SWHAM), which resamples the raw data by applying the serial tempering (ST) protocol; the multi-state distributions can be obtained directly by running replica exchange-like SWHAM (RE-SWHAM), which resamples the raw data by applying the replica exchange (RE) protocol. If the simulations at some states are far from convergence, the multi-state distributions can be estimated by Stratified RE-SWHAM. Local WHAM^22^, which is a variant of ST-SWHAM that couples the adjacent states by a stochastic resampling procedure, is also included in this software package. The remaining part of the paper proceeds as follows: First, we briefly review the theoretical basis of UWHAM and SWHAM. Then we introduce the tutorial examples on the web page of the UWHAM and SWHAM software package.

## Methods and Discussion

### UWHAM

Suppose *M* parallel (independent or coupled) simulations in the canonical ensemble are run at *M* states. Each state is characterized by a specific combination of thermodynamic parameters and potential energy functions. They are referred to as *λ*-states in the remaining part of this paper to avoid the confusion with the terms such as conformational states and microstates. Suppose *X*_*αi*_ is the *i*th microstate observed at the *α*th *λ*-state, the probability of observing *X*_*αi*_ at the *γ*th *λ*-state is

1 $${P}_{\gamma }({X}_{\alpha i}) \sim \frac{{q}_{\gamma }({\{x\}}_{\alpha i})}{{Z}_{\gamma }}=\frac{\exp \{\,-\,{\beta }_{\gamma }{E}_{\gamma }(\{x{\}}_{\alpha i})\}}{{Z}_{\gamma }},$$

where *q*_*γ*_({*x*}_*αi*_) = exp {−*β*_*γ*_*E*_*γ*_({*x*}_*αi*_)} is the Boltzmann’s factor of *X*_*αi*_ at the *γ*th *λ*-state; {*x*}_*αi*_ is the coordinates of the microstate *X*_*αi*_; *β*_*γ*_ is the inverse temperature of the *γ*th *λ*-state; *E*_*γ*_({*x*}_*αi*_) is the potential energy of the microstate *X*_*αi*_ at the *γ*th *λ*-state; and *Z*_*γ*_ is the partition function of the *γ*th *λ*-state. The likelihood of the observed data is proportional to^14^

2 $$\prod _{\alpha =1}^{M}\prod _{i=1}^{{N}_{\alpha }}\frac{{\rm{\Omega }}({u}_{\alpha i}){q}_{\alpha }({u}_{\alpha i})}{{Z}_{\alpha }},$$

where *u*_*αi*_ is the energy coordinate of the microstate *X*_*αi*_ that in general may be written as the sum of a reference energy plus perturbations (see ref.^14^). *N*_*α*_ is the total number of observations observed at the *α*th *λ*-state; and Ω(*u*_*αi*_) is the density of states. Let ${\hat{Z}}_{\alpha }$ and $\hat{{\rm{\Omega }}}({u}_{\gamma i})$ denote estimates of the partition function of the *α*th *λ*-states and the density of states of *u*_*γi*_, respectively. These two estimates satisfy the equation

3 $${\hat{Z}}_{\alpha }=\sum _{\gamma =1}^{M}\,\sum _{i=1}^{{N}_{\gamma }}\,{q}_{\alpha }({u}_{\gamma i})\hat{{\rm{\Omega }}}({u}_{\gamma i}\mathrm{)}.$$

Maximizing the log likelihood function yields

4 $$\hat{{\rm{\Omega }}}({u}_{\gamma i})=\frac{1}{\sum _{\kappa =1}^{M}\,{N}_{\kappa }{\hat{Z}}_{\kappa }^{-\,1}\,{q}_{\kappa }({u}_{\gamma i})}.$$

Eqs (3) and (4) are the UWHAM (or MBAR) equations^13,14^. Note that the UWHAM estimates do not depend on the original *λ*-state at which each observation was observed. Therefore the UWHAM equations can be simplified as

5 $$\begin{array}{rcl}{\hat{Z}}_{\alpha } & = & \sum _{i=1}^{N}\,{q}_{\alpha }({u}_{i})\hat{{\rm{\Omega }}}({u}_{i})\\ \hat{{\rm{\Omega }}}({u}_{i}) & = & \frac{1}{\sum _{\kappa =1}^{M}\,{N}_{\kappa }{\hat{Z}}_{\kappa }^{-\,1}\,{q}_{\kappa }({u}_{i})},\end{array}$$

where $N={\sum }_{\alpha =1}^{M}\,{N}_{\alpha }$ is the total number of observations.

The UWHAM estimate of the probability of observing the observation *u*_*i*_ at the *α*th *λ*-state is

6 $${\hat{p}}_{\alpha }({u}_{i})={\hat{Z}}_{\alpha }^{-\,1}\hat{{\rm{\Omega }}}({u}_{i}){q}_{\alpha }({u}_{i})=\frac{{\hat{w}}_{\alpha }({u}_{i})}{{\hat{Z}}_{\alpha }}.$$

where ${\hat{w}}_{\alpha }({u}_{i})=\hat{{\rm{\Omega }}}({u}_{i}){q}_{\alpha }({u}_{i})$ is the unnormalized probability. We can define one of the *λ*-states as the reference state, and the normalized probability of observing the observation *u*_*i*_ at the reference state is

7 $${\hat{w}}_{0}({u}_{i})=\frac{\hat{{\rm{\Omega }}}({u}_{i}){q}_{0}({u}_{i})}{\sum _{j=1}^{N}\,\hat{{\rm{\Omega }}}({u}_{j}){q}_{0}({u}_{j})};$$

and ${\hat{w}}_{\alpha }={\hat{w}}_{0}({u}_{i}){\rm{\Delta }}{q}_{\alpha }({u}_{i})$, where Δ*q*_*α*_(*u*_*i*_) = *q*_*α*_(*u*_*i*_)/*q*_0_(*u*_*i*_) = exp{−[*β*_*α*_*E*_*α*_(*u*_*i*_) − *β*_0_*E*_0_(*u*_*i*_)]} is the biasing factor. Then the equation array Eq. (5) can be rewritten as

8 $$\begin{array}{rcl}{\hat{Z}}_{\alpha } & = & \sum _{i=1}^{N}\,{\hat{w}}_{0}({u}_{i}){\rm{\Delta }}{q}_{\alpha }({u}_{i})\\ {\hat{w}}_{0}({u}_{i}) & = & \frac{1}{\sum _{\kappa =1}^{M}\,{N}_{\kappa }{\hat{Z}}_{\kappa }^{-\,1}{\rm{\Delta }}{q}_{\kappa }({u}_{i})}.\end{array}$$

In practice, the UWHAM program solves the equation array Eq. (8) instead of Eq. (5). Suppose *A* is a property of interest of the system. According to Eq. (6), the expectation value of the property *A* at the *α*th *λ*-state is calculated by the weighted average

9 $${\langle A\rangle }_{\alpha }=\frac{\sum _{i=1}^{N}\,{\hat{w}}_{\alpha }({u}_{i})A({u}_{i})}{\sum _{i=1}^{N}\,{\hat{w}}_{\alpha }({u}_{i})}=\frac{\sum _{i=1}^{N}\,{\hat{w}}_{0}({u}_{i}){\rm{\Delta }}{q}_{\alpha }({u}_{i})A({u}_{i})}{\sum _{i=1}^{N}\,{\hat{w}}_{0}({u}_{i}){\rm{\Delta }}{q}_{\alpha }({u}_{i})}.$$

where *A*(*u*_*i*_) is the the property *A* measured by using the *i*th observation.

Currently, a self-consistent iteration solver and a solver that optimizes a convex function by using the Newton-Raphson algorithm^14^ have been implemented in the UWHAM program to solve the UWHAM equations.

### SWHAM

Suppose the raw data were generated from simulations at *M λ*-states, and the total number of observations is *N*. During the procedure of UWHAM analysis, the program needs to evaluate *M* biasing factors (or Boltzmann’s factors) for each observation at the beginning. Namely, the UWHAM program evaluates a biasing matrix which contains *n* × *M*^2^ elements, where *n* = *N*/*M* is the average number of observations observed at each *λ*-state. Then the UWHAM equations are solved by minimization of a convex function, which involves multiplication of matrices that contain *M* × *N* elements (as large as the biasing matrix) and diagonalization of matrices that contain *M* × *M* elements. The costs of memory and computational time of running UWHAM are proportional to the second order of the number of *λ*-states *M*. To remove this computational bottleneck in scaling up UWHAM, we developed methods which solve UWHAM equations stochastically by using the generalized ensemble algorithms.

#### RE-SWHAM

**Figure 1:**
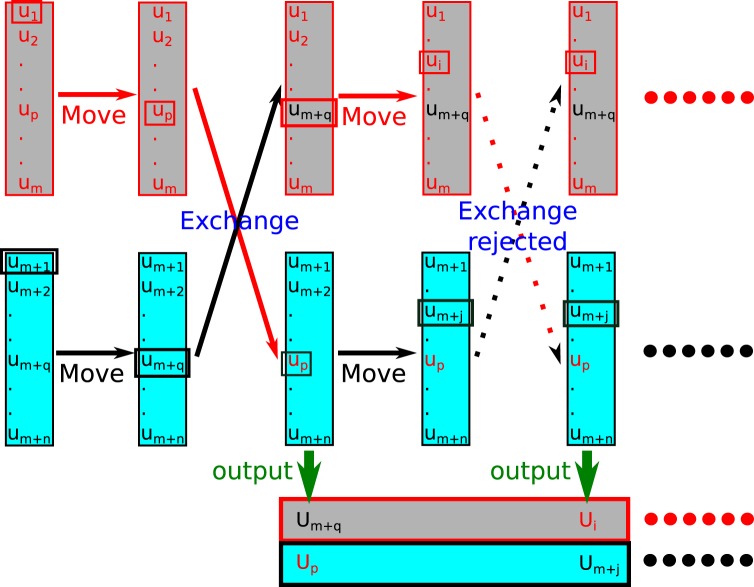
An illustration of the RE-SWHAM algorithm. This drawing illustrates two replica exchange cycles of the RE-SWHAM method, and shows only two *λ*-states with “gray” or “cyan” color. In each cycle one data element is chosen from *λ*-state first, then a replica exchange is performed. In the first cycle since the swap is accepted, the data associated with the two replicas is swapped to the other *λ*-state’s data array. At the end of each cycle, the data associated with replicas are recorded as the output like explicit RE simulations. Reprinted (adapted) with permission from ref.^21^. Copyright (2015) American Chemical Society.

RE-SWHAM is an algorithm that we developed to solve the UWHAM equations stochastically by applying the replica exchange (RE) protocol to resample the raw data generated by multi-state simulations^21^. As shown in Fig. 1, the observations observed at each *λ*-state are collected as the database for that *λ*-state beforehand. Then RE-SWHAM analyses are run by performing cycles of RE simulations. Each cycle consists of a “move” procedure and an “exchange” procedure. During the move procedure of RE-SWHAM, an observation in the database of a *λ*-state is randomly chosen with equal probability to associate with the replica at that *λ*-state. During the exchange procedure of RE-SWHAM, we attempt to swap two random replicas based on the Metropolis criterion. If the swap is accepted, in addition to swapping the replicas, the observation associated with the replica is also swapped to the database of the other *λ*-state^21^. The exchange step should be repeated multiple times to approach the infinite swapping limit for the best sampling efficiency^24^. At the end of the exchange procedure, the observation associated with the replica at each *λ*-state is recorded as the output of that *λ*-state. Note the direct outputs of RE-SWHAM are the estimates of the equilibrium distribution at each *λ*-state.

In ref.^21^, we proved that the distribution of the outputs of RE-SWHAM at each *λ*-state are asymptotic to the UWHAM estimate when the number of observations observed at each *λ*-state is large by treating RE-SWHAM as a random walk in the space of the weight arrays of observations. Here we provide an alternative proof. Consider a trial exchange in RE-SWHAM which swaps one observation *u*_*m*_ at the *α*th *λ*-state and the other observation *u*_*n*_ at the *γ*th *λ*-state. The probability that this trial exchange is accepted is

10 $$\begin{array}{rcl}{P}_{ex} & = & {\tilde{p}}_{\alpha }({u}_{m}){\tilde{p}}_{\gamma }({u}_{n})\,{\rm{\min }}\,(1,\frac{\exp \,[\,-\,{\beta }_{\alpha }{E}_{\alpha }({u}_{n})]\,\exp \,[\,-\,{\beta }_{\gamma }{E}_{\gamma }({u}_{m})]}{\exp \,[\,-\,{\beta }_{\alpha }{E}_{\alpha }({u}_{m})]\,\exp \,[\,-\,{\beta }_{\gamma }{E}_{\gamma }({u}_{n})]})\\  & = & {\tilde{p}}_{\alpha }({u}_{m}){\tilde{p}}_{\gamma }({u}_{n}){\rm{\Psi }}(\mathrm{log}\,[\frac{{q}_{\alpha }({u}_{m}){q}_{\gamma }({u}_{n})}{{q}_{\alpha }({u}_{n}){q}_{\gamma }({u}_{m})}])\,\end{array}.$$

where ${\tilde{p}}_{X}({u}_{Y})$ is the normalized time-average probability of choosing the observation *u*_*Y*_ to associate with the replica at the *X* th *λ*-state, and Ψ is the Metropolis function^25^

11 $${\rm{\Psi }}(x)=\,{\rm{\min }}\,\mathrm{(1},\exp \,[\,-\,x]),$$

which has the property Ψ(*x*)/Ψ(−*x*) = exp{−*x*}. Consider the reverse trial exchange that swaps the observation *u*_*n*_ at the *α*th *λ*-state and the observation *u*_*m*_ at the *γ λ*-state. The probability that this trial exchange is accepted is

12 $$\begin{array}{rcl}{P^{\prime} }_{ex} & = & {\tilde{p}}_{\alpha }({u}_{n}){\tilde{p}}_{\gamma }({u}_{m})\,{\rm{\min }}\,(1,\frac{\exp \,[\,-\,{\beta }_{\alpha }{E}_{\alpha }({u}_{m})]\,\exp \,[\,-\,{\beta }_{\gamma }{E}_{\gamma }({u}_{n})]}{\exp \,[\,-\,{\beta }_{\alpha }{E}_{\alpha }({u}_{n})]\,\exp \,[\,-\,{\beta }_{\gamma }{E}_{\gamma }({u}_{m})]})\\  & = & {\tilde{p}}_{\alpha }({u}_{n}){\tilde{p}}_{\gamma }({u}_{m}){\rm{\Psi }}(\mathrm{log}[\frac{{q}_{\alpha }({u}_{n}){q}_{\gamma }({u}_{m})}{{q}_{\alpha }({u}_{m}){q}_{\gamma }({u}_{n})}])\,.\end{array}$$

If the RE-SWHAM resampling procedure converges, *P*_*ex*_ and *P*′_*ex*_ will agree with each other for each pair of observations (*u*_*m*_, *u*_*n*_) and each pair of *λ*-states (*α*, *γ*), which leads to the detailed balance relation of RE-SWHAM:

13 $$\frac{{\tilde{p}}_{\alpha }({u}_{m})/{q}_{\alpha }({u}_{m})}{{\tilde{p}}_{\alpha }({u}_{n})/{q}_{\alpha }({u}_{n})}=\frac{{\tilde{p}}_{\gamma }({u}_{m})/{q}_{\gamma }({u}_{m})}{{\tilde{p}}_{\gamma }({u}_{n})/{q}_{\gamma }({u}_{n})},\,{\rm{for}}\,{\rm{all}}\,({u}_{m},{u}_{n})\,{\rm{and}}\,({\boldsymbol{\alpha }},\gamma ).$$

Eq. (13) can be rewritten as

14 $$\frac{{\tilde{p}}_{\alpha }({u}_{m})/{q}_{\alpha }({u}_{m})}{{\tilde{p}}_{0}({u}_{m})/{q}_{0}({u}_{m})}=\frac{{\tilde{p}}_{\alpha }({u}_{n})/{q}_{\alpha }({u}_{n})}{{\tilde{p}}_{0}({u}_{n})/{q}_{0}({u}_{n})}={\hat{Z}}_{\alpha }^{-\,1},\,{\rm{for}}\,{\rm{all}}\,({u}_{m},{u}_{n})\,{\rm{and}}\,{\boldsymbol{\alpha }}.$$

where subscript 0 denotes the reference state. Then the probability ${\tilde{p}}_{\alpha }({u}_{m})$ can be expressed as

15 $${\tilde{p}}_{\alpha }({u}_{m})={\hat{Z}}_{\alpha }^{-\,1}\hat{{\rm{\Omega }}}({u}_{m}){q}_{\alpha }({u}_{m}),\,{\rm{for}}\,{\rm{all}}\,{u}_{m}\,{\rm{and}}\,{\boldsymbol{\alpha }},$$

where $\hat{{\rm{\Omega }}}({u}_{m})={\tilde{p}}_{0}({u}_{m})/{q}_{0}({u}_{m})$. Summing both sides of Eq. (15) over all the observations and applying the relationship ${\sum }_{m=1}^{N}\,{\tilde{p}}_{\alpha }({u}_{m})=1$ at each *λ*-state yields

16 $${\hat{Z}}_{\alpha }=\sum _{m=1}^{N}\,\hat{{\rm{\Omega }}}({u}_{m}){q}_{\alpha }({u}_{m}\mathrm{)}.$$

Note that the probability of finding the observation *u*_*m*_ in the database of the *α*th *λ*-state is ${N}_{\alpha }\,{\tilde{p}}_{\alpha }({u}_{m})$ and there is one copy of each observation in the databases of all *λ*-states, namely, ${\sum }_{\alpha =1}^{M}\,{N}_{\alpha }\,{\tilde{p}}_{\alpha }({u}_{m})=1$. Multiplying both side of Eq. (15) by *N*_*α*_ and summing over all the *λ*-states yields

17 $$\hat{{\rm{\Omega }}}({u}_{m})=\frac{1}{\sum _{\alpha \mathrm{=1}}^{M}\,{N}_{\alpha }\,{\hat{Z}}_{\alpha }^{-\,1}{q}_{\alpha }({u}_{m})}.$$

Thus, the RE-SWHAM estimates ${\hat{Z}}_{\alpha }$ and $\hat{{\rm{\Omega }}}({u}_{m})$ satisfy Eqs (16) and (17), which are equivalent to the UWHAM equations (Eq. (5)).

#### ST-SWHAM

**Figure 2:**
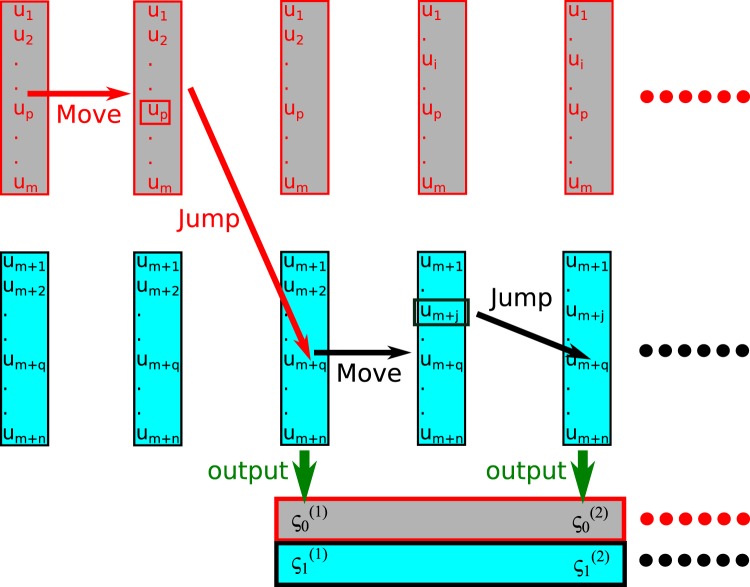
An illustration of the ST-SWHAM algorithm. This drawing illustrates two serial tempering cycles of the ST-SWHAM method, and shows only two *λ*-states with “gray” or “cyan” color. In each cycle one data element is chosen from the *λ*-state sampled by the replica with equal probability to associate with the replica. Then the replica jumps to one of the *λ*-states according to the probability calculated by Eq. [18]. At the end of each cycle, the free energy estimates {*ζ*_*k*_} are adjusted to match the observed proportion of the replica being at the *κ*th *λ*-state *π*_*κ*_ with the proportion of the *κ*th *λ*-state of the raw data generated by the multi-state simulations ${\pi }_{\kappa }^{0}$.

ST-SWHAM is an algorithm that we developed to solve the UWHAM equations stochastically by applying the serial tempering (ST) protocol to resample the raw data generated by multi-state simulations^22^. The procedure is illustrated in Fig. 2. Like the RE-SWHAM analysis, the observations observed at each *λ*-state are collected as the database for that *λ*-state beforehand. However, unlike resampling the data using replica exchanges, there is only one “simulation run” in the serial tempering resampling algorithm. For the sake of comparison and convenience, we still refer to this single simulation as a replica in this paper. Serial tempering simulations are also run by cycles, and each cycle consists of a “move” procedure and a “jump” procedure. During the move procedure of ST-SWHAM, an observation in the database of the *λ*-state sampled by the replica is randomly chosen with equal probability to associate with the replica. During the jump procedure, the replica jumps to the *α*th *λ*-state according to the probability^22^

18 $$p(\alpha |{u}_{i};\zeta ,{\pi }^{0})=\frac{{\pi }_{\alpha }^{0}{e}^{{\zeta }_{\alpha }}{q}_{\alpha }({u}_{i})}{\sum _{\kappa =1}^{M}\,{\pi }_{\kappa }^{0}{e}^{{\zeta }_{\kappa }}{q}_{\kappa }({u}_{i})},$$

where *u*_*i*_ is the *i*th observation associated with the replica; *ζ*_*κ*_ = −ln*Z*_*κ*_ is the unitless free energy of the *κ*th *λ*-state; ${\pi }_{\kappa }^{0}={N}_{\kappa }/N$ is the proportion of the *κ*th *λ*-state of the raw data generated by the multi-state simulations; and *q*_*κ*_(*u*_*i*_) is the biasing factor of the *i*th observation at the *κ*th *λ*-state. Suppose *π*_*κ*_ is the observed proportion of the *κ*th *λ*-state sampled by the replica during the ST-SWHAM analysis. The values of {*ζ*_*κ*_} are adjusted during the analysis of ST-SWHAM until the observed proportion of the replica being at the *κ*th *λ*-state *π*_*κ*_ agrees with ${\pi }_{\kappa }^{0}$^22^. Note the direct outputs of ST-SWHAM are the estimates of the free energies of different *λ*-states — {*ζ*_*κ*_}.

It can be shown that *ζ*_*κ*_ is the UWHAM estimate of the free energy of the *κ*th *λ*-state when *π*_*κ*_ and ${\pi }_{\kappa }^{0}$ agree with each other for all *λ*-states. The details of the proof that ST-SWHAM solves the UWHAM equations stochastically can be found in ref.^22^. One brief rationale is as follows. First, if *π*_*κ*_ equals ${\pi }_{\kappa }^{0}$, the probability of each observation being chosen to associate with the replica during the ST-SWHAM analysis is 1/*N*, where *N* is the total number of observations. Therefore, the observed proportion of the *α*th *λ*-state sampled by the replica during the ST-SWHAM analysis is

19 $${\pi }_{\alpha }=\frac{1}{N}\sum _{i=1}^{N}\,p(\alpha |{u}_{i};\zeta ,{\pi }^{0})=\frac{1}{N}\sum _{i=1}^{N}\,\frac{{\pi }_{\alpha }^{0}{e}^{{\zeta }_{\alpha }}{q}_{\alpha }({u}_{i})}{\sum _{\kappa =1}^{M}\,{\pi }_{\kappa }^{0}{e}^{{\zeta }_{\kappa }}{q}_{\kappa }({u}_{i})}.$$

On the other hand, note that

20 $$\frac{1}{N}\sum _{i=1}^{N}\,\frac{{e}^{{\hat{\zeta }}_{\alpha }}{q}_{\alpha }({u}_{i})}{\sum _{\kappa =1}^{M}\,{\pi }_{\kappa }^{0}{e}^{{\hat{\zeta }}_{\kappa }}{q}_{\kappa }({u}_{i})}=\sum _{i=1}^{N}\,\frac{\hat{{\rm{\Omega }}}({u}_{i}){q}_{\alpha }({u}_{i})}{{Z}_{\alpha }}=\sum _{i=1}^{N}\,{\hat{p}}_{\alpha }({u}_{i})=1.$$

Then ${\pi }_{\alpha }^{0}$ can be rewritten as

21 $${\pi }_{\alpha }^{0}=\frac{1}{N}\sum _{i=1}^{N}\,\frac{{\pi }_{\alpha }^{0}{e}^{{\hat{\zeta }}_{\alpha }}{q}_{\alpha }({u}_{i})}{\sum _{\kappa =1}^{M}\,{\pi }_{\kappa }^{0}{e}^{{\hat{\zeta }}_{\kappa }}{q}_{\kappa }({u}_{i})}.$$

Comparison between Eqs (19) and (21) leads to the conclusion that *π*_*α*_ and ${\pi }_{\alpha }^{0}$ agree with each other if ${\zeta }_{\alpha }={\hat{\zeta }}_{\alpha }$ for each *λ*-state.

The jump of the replica following Eq. (18) was referred to as the global jump proposal in ref.^22^ because the replica can reach any *λ*-state of the system by one jump. According to Eq. (18), every jump of the replica requires calculations of *M* exponential functions, where *M* is the total number of *λ*-states. When the total number of states is large, ST-SWHAM analyses using the global jump proposal take a long time to converge. In our software package, we implemented a much faster approximate solver of UWHAM–ST-SWHAM using a local jump proposal. This algorithm was referred to as local WHAM in ref.^22^ because the replica can only be at the *λ*-states that are the local neighbors of the initial *λ*-state at the end of the jump procedure if the number of jumps per cycle is finite. Suppose the replica that associates with the observation *u*_*i*_ is at the *γ*th *λ*-state initially. The procedure of performing one jump in local WHAM is as follows^22^:

- select a trial *λ*-state with uniform probabilities from the nearest neighbors of the *γ*th *λ*-state, suppose the chosen *λ*-state is the *α*th *λ*-state.
- accept the *α*th *λ*-state as the new *λ*-state to jump to according to the Metropolis probability

22 $${\rm{\min }}\{1,\,\frac{{\rm{\Gamma }}(\alpha ,\gamma )}{{\rm{\Gamma }}(\gamma ,\alpha )}\,\frac{p(\alpha |{u}_{i};\zeta ,{\pi }^{0})}{p(\gamma |{u}_{i};\zeta ,{\pi }^{0})}\},$$

where *p*(*α*|*u*_*i*_; *ζ*, *π*^0^) and *p*(*γ*|*u*_*i*_; *ζ*, *π*^0^) are defined by Eq. (18); and Γ(*γ*, *α*) is the probability of choosing the *α*th *λ*-state as the trial *λ*-state when the replica is at the *γ*th *λ*-state originally. Namely, Γ(*γ*, *α*) = 1/*n*_*γ*_, where *n*_*γ*_ is the total number of the nearest neighbors of the *γ*th *λ*-state if the *α*th *λ*-state is one of the nearest neighbors of the *γ*th *λ*-state; Γ(*γ*, *α*) = 0 otherwise.

As can be seen, the replica can only be at the original *λ*-state or one of its nearest neighbors after one jump. However, the replica can diffuse further away from the original *λ*-state by repeating this one jump procedure multiple times. As the number of jumps per cycle increases, the results of local WHAM converges asymptotically to the results of ST-SWHAM that uses the global jump proposal^22^. Therefore, the jump of the replica following Eq. (18) in the infinite jump limit in serial tempering simulations is analogous to the infinite swapping limit in replica exchange simulations^24^.

In ST-SWHAM, the free energy estimates are adjusted during the analysis until the observed proportion of the replica being at the *κ*th *λ*-state *π*_*κ*_ agrees with the proportion of the *κ*th *λ*-state of the raw data generated by the multi-state simulations ${\pi }_{\kappa }^{0}$. So far a variant of the updating algorithm discussed in ref.^22^ is implemented in the ST-SWHAM program.

### Stratified RE-SWHAM

When applying UWHAM and its stochastic solvers, the basic assumption is that the simulation at each *λ*-state is “approximately” equilibrated. However, this assumption might not always hold. To handle such situations, we developed an analysis tool called Stratified-UWHAM and its stochastic solver Stratified RE-SWHAM to compute free energy and expectations from a multi-state ensemble when the simulations at a subset of *λ*-states are far from global equilibrium^23^. In ref.^23^, we showed that the Stratified UWHAM equations can be solved in the form of the original UWHAM equations (Eq. (5)) with an expanded set of *λ*-states. The stochastic solver, Stratified RE-SWHAM, has been included in the UWHAM and SWHAM software package. See the Supporting Information for a brief review and discussion about Stratified UWHAM and Stratified RE-SWHAM.

### Illustrative applications

So far the tutorial examples include how to analyze the data generated by “one dimensional umbrella sampling”, “two dimensional umbrella sampling”, “temperature replica exchange”, “Hamiltonian replica exchange”, “two dimensional replica exchange” and “ free energy perturbation” simulations. The tutorials provide the raw data generated by different types of multi-state simulations and explain the corresponding analysis procedures and outputs in details.

### One Dimensional Umbrella Sampling

We explain how to apply UWHAM or ST-SWHAM to analyze the raw data generated by one dimensional umbrella sampling simulations. The potential function of the system studied in this example is a one dimensional double well potential^26^

23 $$U(x)=\frac{H}{{W}^{4}}{({x}^{2}-{W}^{2})}^{2},$$

where *H* = 20 *k*_*B*_*T* is the height of the barrier between the two wells; *k*_*B*_ is Boltzmann’s constant; *T* is the temperature; and *W* = 1 is the half width between the two minima of the potential. This one dimensional potential can be explored by a Brownian particle simulated with the over-damped Langevin dynamics^26^. Here we applied 31 parabolic potentials in the region between *x* = −3 to *x* = 3 to perform the umbrella sampling simulations. Then UWHAM and ST-SWHAM are used to analyze the data and construct the potential energy profile.

Umbrella sampling simulations are usually applied to construct free energy profiles for systems with multiple degrees of freedom. Although the example that we used here is a Brownian particle governed by a one dimensional potential function, the analysis procedure is the same for applying UWHAM or ST-SWHAM to construct one dimensional free energy profiles of complex systems. In such cases, the position of the complex system projected on the chosen reaction coordinate is analogous to the position of the Brownian particle in this tutorial.

### Two Dimensional Umbrella Sampling

This example explains how to apply UWHAM or ST-SWHAM to raw data generated by two dimensional umbrella sampling simulations (of ~100 degrees of freedom) to construct the free energy profile. The system studied in this example is an alanine dipeptide (AlaD) molecule in implicit solvent at 300 *K*. The simulations were performed by using the GROMACS 5.1.2 simulation package with the Amber99SB force field and the OBC GB model^27,28^. To explore the two dimensional free energy surface (the Ramachandran plot of AlaD), we applied 24 × 24 parabolic potentials by using the PLUMED plugin^29^ to perform the umbrella sampling simulations. The Ramachandran plots of AlaD are constructed by using the UWHAM and ST-SWHAM estimates.

### Temperature Replica Exchange

We explain how to apply UWHAM or RE-SWHAM to raw data generated by temperature replica exchange simulations to obtain the estimates of the equilibrium distribution at the *λ*-state of interest. The system studied in this example is the same as the previous example–an alanine dipeptide (AlaD) molecule in implicit solvent. The RE simulations were performed by using the GROMACS 5.1.2 simulation package with the Amber99SB force field and the OBC GB model^27,28^. The coupled simulations were run at 10 temperatures (300 *K*, 317.52 *K*, 336.063 *K*, 355.689, 376.462 *K*, 398.447 *K*, 421.716 *K*, 446.345 *K*, 472.411, 500 *K*). The Ramachandran plots of AlaD in implicit solvent at 300 *K* are constructed by using the UWHAM and RE-SWHAM estimates.

### Free Energy Perturbation

This example shows how to analyze the data generated by free energy perturbation (FEP) simulations. Here we calculate the solvation free energy of a water molecule in pure solvent (TIP3P) at 300 *K* by using the slow-growth method. The simulations were performed by using the GROMACS 5.1.2^27^ and the TIP3P water model. In this example, we ran 11 independent parallel simulations for a box of pure solvent with a fixed tagged water molecule inside. The interaction between the tagged water molecule and the environment were gradually turned off through 11 *λ*-states^30^. The chosen *λ* values are 0.0, 0.1, 0.2, 0.3, 0.4, 0.5, 0.6, 0.7, 0.8, 0.9, 1.0. The UWHAM estimate of the solvation free energy of a water molecule in pure solvent is −6.18 *kcal*/*mol*. One can obtain the same result by using ST-SWHAM. More details and discussion about measuring the excess chemical potential of water molecules in solution using UWHAM can be found in ref.^30^.

### BEDAM: Hamiltonian Replica Exchange

We explain how to use UWHAM or RE-SWHAM to analyze the data generated by Hamiltonian replica exchange simulations. In this example, we study the binding affinity of a guest molecule (heptanoate) to a host molecule (*β*-cyclodextrin) in implicit solvent (OPLA-AA/AGBNP2)^31,32^. Here we apply the binding energy distribution analysis method (BEDAM)^33^ to obtain the binding free energy and binding energy distributions of this complex. BEDAM is a free energy method based on the Hamiltonian replica exchange algorithm. Suppose there are *M* parallel simulations in BEDAM, the Hamiltonian (potential) function of the *i*th *λ*-state is

24 $${H}_{i}={V}_{0}+{\lambda }_{i}u,$$

where *V*_0_ is the effective potential energy of the complex without the direct and solvent-mediated ligand-receptor interactions, and *u* is the binding energy^33^. Namely, the *λ* factor in BEDAM simulations linearly scales the interaction between the ligand and acceptor. We ran BEDAM simulations at 300 *K* by using 16 *λ*-states. The chosen *λ* values are 0.0, 0.001, 0.002, 0.004, 0.01, 0.04, 0.07, 0.1, 0.2, 0.4, 0.6, 0.7, 0.8, 0.9, 0.95, 1.0^21^. We applied UWHAM to estimate the binding free energy of the *β*-cyclodextrin Heptanoate Complex— −0.603 *kcal*/*mol* + *G*_*vsite*_, where *G*_*vsite*_ is a correction because of the restraint applied to the ligand during the BEDAM simulation. One can obtain the same result by applying ST-SWHAM to the raw data. We also show how to apply UWHAM or RE-SWHAM to estimate the equilibrium distribution of the binding energy at the *λ* = 1 state (full interaction state).

### Two Dimensional (Temperature and Hamiltonian) Replica Exchange

This example shows how to use SWHAM to analyze the data generated by two dimensional (Hamiltonian and temperature) replica exchange simulations. We study the binding affinity of a guest molecule (heptanoate) to a host molecule (*β*-cyclodextrin) in implicit solvent (OPLA-AA/AGBNP2)^32^ at different temperatures. The raw data used in this example were generated by 15 separated BEDAM^33^ simulations at temperatures 200 *K*, 206 *K*, 212 *K*, 218 *K*, 225 *K*, 231, 238 *K*, 245 *K*, 252 *K*, 260 *K*, 267 *K*, 275, 283 *K*, 291 *K*, 300 *K*. The chosen *λ* values are the same as the previous example–0.0, 0.001, 0.002, 0.004, 0.01, 0.04, 0.07, 0.1, 0.2, 0.4, 0.6, 0.7, 0.8, 0.9, 0.95, 1.0. There are totally 16 × 15 = 240 states, and each state has 144,000 data points^21,22^. Although there are no exchanges between replicas at different temperatures, the procedure described in this tutorial can be applied to two dimensional (Hamiltonian and temperature) replica exchange simulations without any alteration. The goal of this practice is to obtain the best estimates of the binding affinity at 200 *K*, which is the most difficult for BEDAM simulation to converge. Because the raw data ensemble is large, UWHAM is not suitable to analyze them directly. Here, we applied RE-SWHAM to estimate the equilibrium distribution of binding energies of each *λ*-state at 200 *K*. And the RE-SWHAM results are compared with the corresponding distributions calculated from the raw data. See ref.^21^ and ^22^ for more discussion about this tutorial example. The equilibrium distributions constructed by the RE-SWHAM output can be used as the input for UWHAM to estimate the binding free energy at the temperature of interest. The binding free energy of the *β*-cyclodextrin Heptanoate Complex is about −6.3 *kcal*/*mol* + *G*_*vsite*_ at 200 *K*, which is much stronger compared with its binding free energy at 300 *K*. This result can also be obtained by applying ST-SWHAM with the local jump algorithm to the raw data directly.

### Two Binding Modes of the *β*-cyclodextrin Heptanoate Complex

The *β*-cyclodextrin heptanoate complex has two binding states depending on the orientation of the heptanoate molecule^23^. The two binding modes are referred to as the UP and DOWN macrostates. We ran two sets of independent MD simulations at 300 *K* of the *β*-cyclodextrin heptanoate complex in implicit solvent (AGBNP GB model^32^) at 16 *λ*-states. The initial structures of the complex in the first and the second sets of simulations were chosen from the UP and Down macrostates, respectively. The interaction between the ligand and the receptor was scaled by a *λ* factor like BEDAM^33^, However, all the simulations are independent. The chosen *λ* values are (0.0, 0.001, 0.002, 0.004, 0.01, 0.04, 0.07, 0.1, 0.2, 0.4, 0.6, 0.7, 0.8, 0.9, 0.95, 1.0). In this example, the *λ*-states with the largest seven *λ* values (*λ* = 1.0, 0.95, 0.9, 0.8, 0.7, 0.6, 0.4) are considered as the partially connected states because it is difficult for the binding complex to switch its binding mode and the simulations have not converged at these *λ*-states; the other nine *λ*-states are the fully connected states^23^. This tutorial shows how to apply Stratified RE-SWHAM to estimate the equilibrium distribution at the *λ* = 1 state (full interaction state) when some simulations are far from convergence. See ref.^23^ for more discussion about this tutorial example.

## Supplementary information

Supporting Information

## Data Availability

The UWHAM and SWHAM software package and its tutorials are available from the web page: https://ronlevygroup.cst.temple.edu/software/UWHAM_and_SWHAM_webpage/index.html. The UWHAM and SWHAM software package is distributed using the MIT license. In the future, we will keep adding more examples of the application of UWHAM and SWHAM to the web page. For instance, free energy perturbation (FEP) is one popular method that is applied to measure the relative ligand binding potency^34,35^. Currently we are applying UWHAM to analyze the FEP data and extract a density of states that can be used to estimate the relative binding free energy differences for multiple ligands simultaneously to solve the cycle closure challenge^34^. We will continue optimizing the code and plan to introduce parallelism to the software package.
