# Supplementary material for: The UWHAM and SWHAM Software Package
Source: Sci Rep. 2019 Feb 26;9:2803. doi: 10.1038/s41598-019-39420-x (PMC6391495; doi:10.1038/s41598-019-39420-x)
Supplement: Supplementary file 1 — Supporting Information [file 41598_2019_39420_MOESM1_ESM.pdf]

The UWHAM and SWHAM Software Package: Supporting Information

Bin W. Zhang,* Shima Arasteh, and Ronald M. Levy

E-mail: bin.w.zhang@temple.edu

Stratified UWHAM

The Stratified-UWHAM algorithm is based on coarse-graining. Firstly, we coarse-grained the whole phase space into macrostates, which correspond to energy basins separated by free energy barriers. Then all the λ-states in the system are divided into two groups ($S_1,\ S_2$). For the λ-states in the first group S_1, the simulations are approximately equilibrated among all the macrostates. Namely, all the macrostates are well connected at each λ-state in group S_1. For the λ-states in the second group S_2, the simulations are only locally equilibrated. Namely, the coarse-grained set of macrostates forms a disconnected network of microstate clusters at each λ-state in group S_2.[1]

We assume that the set of observations $\{X_{\alpha i} : i = 1, 2, \cdots N_\alpha\}$ observed at the λ-states in the S_1 group is independently drawn from the distribution

$$P(X_{\alpha i}) \sim \frac{q_\alpha(\{x\}_{\alpha i})}{Z_\alpha} \,\text{for}\, \alpha \in S_1 \,. \tag{1}$$

And the set of observations $\{X_{\alpha i} : X_{\alpha i} \in R_k, i = 1, 2, \cdots N_\alpha\}$ observed at the λ-states in the

*To whom correspondence should be addressed

S_2 group is independently drawn from the distribution

$$P(X_{\alpha i}|(X_{\alpha i} \in R_k)) \sim \frac{q_{\alpha k}(\{x\}_{\alpha i})}{Z_{\alpha k}} \,\mathrm{for}\, \alpha \in S_2\,, \tag{2}$$

where $q_{\alpha k}(\{x\}_{\alpha i}) = q_\alpha(\{x\}_{\alpha i})\delta(\{x\}_{\alpha i} \in R_k)$; $\delta(\{x\}_{\alpha i} \in R_k)$ is the indicator function for a macrostate cluster R_k; and $Z_{\alpha k}$ is the partition function of the kth macrostate cluster at the αth λ-state. The likelihood function of the simulation data for this model is proportional to

$$\prod_{\alpha \in S_2} \prod_k \prod_{i:X_{\alpha i} \in R_k} \left[\frac{q_\alpha(u_{\alpha i})\Omega(u_{\alpha i})}{Z_{\alpha k}}\right] \times \prod_{\alpha \in S_1} \prod_{i=1}^{N_\alpha} \left[\frac{q_\alpha(u_{\alpha i})\Omega(u_{\alpha i})}{Z_\alpha}\right]\,, \tag{3}$$

where $u_{\alpha i}$ is the reduced coordinate of the microstate $X_{\alpha i}$.[1]

In Ref.[1], we showed that the estimating equations from the maximization of Eq.(3) can be solved in the form of UWHAM equations with an expanded set of λ-states. In such case the total number of λ-states of the system increases from $M_1 + M_2 = M$ to $M_1 + \sum_{\alpha=1}^{M_2} K_\alpha$, where M_1 and M_2 are the total numbers of λ-states in the S_1 and S_2 group, respectively; K_α is the number of disconnected macrostate clusters of the αth λ-state in the S_2 group.[1] As mentioned in the main text, the costs of memory and computational time of running UWHAM are proportional to the second order of the total number of λ-states M. It is more computationally expensive to solve the Stratified-UWHAM equations with an expanded set of λ-states.

In Ref[1], we proposed a stochastic solver for the Stratified-UWHAM equations called Stratified RE-SWHAM, which is illustrated in Fig.1. Stratified RE-SWHAM is a variant of RE-WHAM. There are two differences between Stratified RE-SWHAM and the unstratified version. For the stratified variant, when the observations observed at each λ-state are collected as the database for that λ-state, each observation is tagged by the macrostate cluster that it belongs to.[1] The second difference is how to choose an observation from the the database of each λ-state to associate with the replica at that λ-state during the "move" procedure of each replica exchange cycle. For Stratified RE-SWHAM, if the λ-states is in

the S_1 group, we choose one observation from the database of that λ-state with equal probability, which is the same as the original RE-SWHAM. However, for the λ-states in the S_2 group, we choose one observation that is in the same connected macrostate cluster as the previous observation (associated with the replica) from the database of that λ-state with equal probability.[1] In Ref.[1], we proved that the output of Stratified RE-SWHAM at every λ-state is the estimate of the equilibrium distribution of that λ-state.

References

(1) Zhang, B. W.; Deng, N.; Tan, Z.; Levy, R. M. *J. Chem. Theory Comput.* **2017**, *13*, 4660–4674.

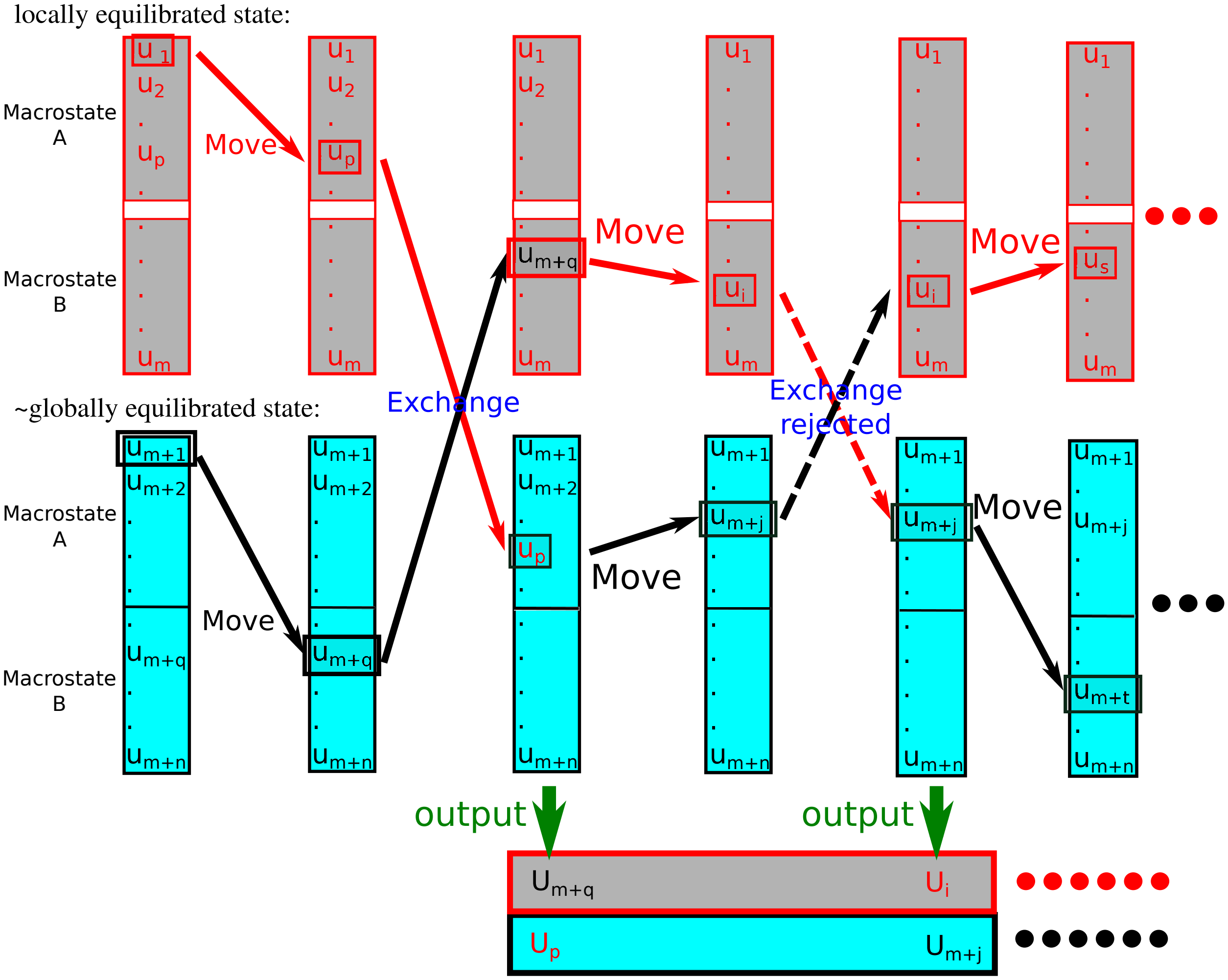

Figure 1: An illustration of the Stratified RE-SWHAM algorithm. This drawing shows two λ-states with "gray" or "cyan" color. Each λ-state has two macrostates A and B. The gray λ-state is locally equilibrated while the simulations at the cyan λ-state are approximately equilibrated among the macrostates. The white gap between macrostates at the gray λ-state represents an uncrossable barrier for the "move" procedure during the Stratified RE-SWHAM analysis. Beforehand, we construct each λ-state a database which contains all the observations obtained from that λ-state, and each observation is tagged by the macrostate which it belongs to. As shown in the picture, the observations are separated into two subgroups A and B. Then Stratified RE-SWHAM is run in cycles, which consists of a "move" procedure and an "exchange" procedure. In the move procedure, Stratified RE-SWHAM chooses an observation to associate with the replica at each λ-state. At the cyan λ-state, the next observation is chosen from the whole database of that λ-state with equal probability. However, at the gray λ-state, the next observation is chosen from the subgroup which the previous observation belongs to with equal probability. In the exchange procedure, if the exchange attempt is accepted, in addition to the swap of the replicas, the observations associated with the replicas are also swapped to the database of the other λ-state. At the end of each cycle, the observation associated with each replica is recorded as the output of Stratified RE-SWHAM. Reprinted (adapted) with permission from Ref[1]. Copyright (2017) American Chemical Society.
